# Toll-Like Receptor 3 Is Involved in Detection of Enterovirus A71 Infection and Targeted by Viral 2A Protease

**DOI:** 10.3390/v10120689

**Published:** 2018-12-05

**Authors:** Kuan-Ru Chen, Chun-Keung Yu, Szu-Hao Kung, Shun-Hua Chen, Chuan-Fa Chang, Tzu-Chuan Ho, Yi-Ping Lee, Hung-Chuan Chang, Lan-Yin Huang, Shih-Yen Lo, Jui-Chung Chang, Pin Ling

**Affiliations:** 1 Institute of Basic Medical Sciences, College of Medicine, National Cheng Kung University, Tainan 70101, Taiwan; dick0119@hotmail.com (K.-R.C.); dckyu@mail.ncku.edu.tw (C.-K.Y.); shunhua@mail.ncku.edu.tw (S.-H.C.); affa@mail.ncku.edu.tw (C.-F.C.); anywayhowtodo@gmail.com (T.-C.H.); yipinglee@hotmail.com (Y.-P.L.); 2 Department of Microbiology and Immunology, College of Medicine, National Cheng Kung University, Tainan 70101, Taiwan; ac430200@gmail.com; 3 Center of Infectious Disease and Signaling Research, College of Medicine, National Cheng Kung University, Tainan 70101, Taiwan; 4 Department of Biotechnology and Laboratory Science in Medicine, National Yang Ming University, Taipei 11221, Taiwan; szkung@ym.edu.tw; 5 Department of Medical Laboratory Science and Biotechnology, College of Medicine, National Cheng Kung University, Tainan 70101, Taiwan; 6 Department of Medicine, College of Medicine, National Cheng Kung University, Tainan 70101, Taiwan; linda_080178@hotmail.com; 7 Department of Laboratory Medicine and Biotechnology, Tzu Chi University, Hualien 97004, Taiwan; losylo@mail.tcu.edu.tw; 8 Department of Prosthetic Dentistry, Chi-Mei Medical Center, Tainan 71004, Taiwan

**Keywords:** Enterovirus 71, Toll-like receptor 3, innate immune system, EV-A71 protease 2A, Type I IFN

## Abstract

Enterovirus A71 (EV-A71) has emerged as a major pathogen causing hand, foot, and mouth disease, as well as neurological disorders. The host immune response affects the outcomes of EV-A71 infection, leading to either resolution or disease progression. However, the mechanisms of how the mammalian innate immune system detects EV-A71 infection to elicit antiviral immunity remain elusive. Here, we report that the Toll-like receptor 3 (TLR3) is a key viral RNA sensor for sensing EV-A71 infection to trigger antiviral immunity. Expression of TLR3 in HEK293 cells enabled the cells to sense EV-A71 infection, leading to type I, IFN-mediated antiviral immunity. Viral double-stranded RNA derived from EV-A71 infection was a key ligand for TLR3 detection. Silencing of TLR3 in mouse and human primary immune cells impaired the activation of IFN-β upon EV-A71 infection, thus reinforcing the importance of the TLR3 pathway in defending against EV-A71 infection. Our results further demonstrated that TLR3 was a target of EV-A71 infection. EV-A71 protease 2A was implicated in the downregulation of TLR3. Together, our results not only demonstrate the importance of the TLR3 pathway in response to EV-A71 infection, but also reveal the involvement of EV-A71 protease 2A in subverting TLR3-mediated antiviral defenses.

## 1. Introduction

Enterovirus A71 (EV-A71) is a positive, single-stranded RNA (ssRNA) virus belonging to the *Enterovirus* genus within the *Picornaviridae* family. EV-A71 infection causes hand, foot, and mouth disease (HFMD) in children and infants [1]. Severe cases of EV-A71 infection are frequently associated with neurological complications and polio-like syndromes, such as encephalitis and meningitis [1]. In recent years, EV-A71 epidemics have been rising in the Asia-Pacific area and beyond [2]. Although EV-A71 vaccines for clinical treatment have been licensed in China [3], better understanding the host-EV-A71 interaction is still critical for gaining insights toward the development of therapeutic strategies against EV-A71 infection [4]. The human scavenger receptor B2 (SCARB2) and P-selectin glycoprotein ligand-1 (PSGL-1) have been shown to serve as viral receptors for EV-A71 infection [5,6]. Studies using human SCARB2 transgenic mice have further confirmed the importance of human SCARB2 for EV-A71 infection in vivo [7,8]. Other cell surface molecules, such as sialylated glycans, nucleolin, and heparan sulfate glycosaminoglycan have also been shown to play a role in enhancing EV-A71 infection on mammalian cells [9,10,11]. Human tryptophanyl-tRNA synthetase has been recently identified to act as an entry receptor for EV-A71 infection [12]. These receptors and molecules play vital roles in the infection and entry of EV-A71 into mammalian cells [13,14]. 

In vertebrates, type I interferons (IFNs) are key cytokines produced by all nuclear cells during virus infection, and they can induce hundreds of IFN-stimulated genes (ISGs) to mount antiviral immunity [15,16]. Studies using murine models have demonstrated the in vivo role of type I IFNs and ISGs in protection against EV-A71 infection [17,18]. Several pattern-recognition receptors in the mammalian innate immune system, including endosomal Toll-like receptors (TLRs), cytosolic RIG-I-like receptors (RLRs), and DNA sensors, have been shown to detect viral nucleic acids during virus infection to trigger the downstream pathways, leading to the production of type I IFNs and inflammatory cytokines [19,20]. By far, TLR3, TLR7, RIG-I, and MDA-5 have been shown to play differential and redundant roles in different cell types to elicit type I IFNs and antiviral immunity against RNA virus infections. Amongst viral RNA sensors, MDA-5 is critical for triggering type I IFN responses upon the infection of picornaviruses, including the encephalomenigitis virus (EMCV) and coxsackievirus B (CVB) [21,22]. Recent evidence has also indicated that MDA5 is involved in detecting EV-A71 RNA to activate IRF3 and IFN-β [23,24]. Other studies using different RNA sensor-deficient mice reveal a critical role for the TLR3-Trif/Ticam-1 pathway in protecting against poliovirus infection [25,26]. 

Cumulative evidence indicates that EV-A71, like other viruses, has evolved strategies to target the viral sensing pathways to counteract type I IFN-mediated antiviral immunity [14,27]. EV-A71 3C protease is shown to target multiple host factors, including RIG-I, Trif, IRF7, CstF-64, and NLRP3 [28,29,30,31,32]. Likewise, EV-A71 2A protease targets IFN-α receptor 1, MDA5, MAVS (also known as IPS-1, VISA, and Cardif), IFN-γ-induced STAT1 serine phosphorylation, and NLRP3 [32,33,34,35,36]. Even though emerging evidence has demonstrated the intricate interactions between EV-A71 and the mammalian innate immune system [37], our understanding of how the host viral RNA sensors detect EV-A71 infection to trigger type I IFN-mediated antiviral responses is still quite lacking. Our current work is an attempt to address this critical issue further.

## 2. Materials and Methods

### 2.1. Ethics Statement

In this study, human monocytes were purified from leukocyte-rich buffy coats obtained from healthy blood donors (Tainan Blood Center, Tainan, Taiwan). There was a cooperation protocol between our Institution and the Tainan Blood Center, where blood donations allowed the use of these products for study purposes. This study was approved by the Institutional Review Board of National Cheng Kung University Hospital (NCKUH) (IRB No.: ER-10-212) and adhered to the Declaration of Helsinki. All experiments relating to humans were carried out in accordance with the approved guidelines and regulations. All participants provided informed written consent before blood donations. All animal protocols were approved by the Institutional Animal Care and User Committee (NCKU-IACUC-103146) at National Cheng Kung University, and all animal experiments were performed in accordance with the approved guidelines and regulations.

### 2.2. Cells, Viruses, and Reagents

Human embryonic kidney 293 (HEK293) cells, HEK293T, and the Vero cell were described previously [38]. Rhabdomyosarcoma (RD) and SK-N-SH cells were grown in DMEM supplemented with 10% fatal bovine serum (FBS, Hyclone), 1% penicillin, and streptomycin. To prepare mouse bone marrow-derived macrophages (BMMs), bone marrow cells isolated from 8- to 12-week-old C57BL/6 mice were cultured in DMEM containing 10% fatal bovine serum, M-CSF 20 ng/mL (PeproTech), and 1% penicillin and streptomycin for 6 days. To prepare human monocyte-derived dendritic cells (MoDCs), monocytes were purified from leukocyte-rich buffy coats obtained from healthy blood donors (Tainan Blood Center, Tainan, Taiwan). Peripheral blood mononuclear cells were isolated by a Ficoll-Paque Plus (GE Healthcare) gradient centrifugation, and cultured in a 75 cm^2^ tissue culture flask for 2 h at 37 °C in RPMI 1640. After 2 h, non-adherent cells were removed by washing with cold PBS, and the remaining monocytes were cultured in RPMI 1640 containing 10% FBS, GM-CSF (20 ng/ml, PeproTech), IL-4 (40 ng/ml, PeproTech), 1% penicillin, and streptomycin for 7 days. The EV-A71/Tainan/4643/98 strain (GenBank accession number AF304458) and mouse-adapted strain EV-A71/MP4 were propagated in RD cells, as previously described [39]. The EV-A71 titer was determined by the plaque assay using RD or Vero cells. Influenza A/WSN/33 viruses (H1N1) were described previously [40]. The titer of influenza virus A/WSN/33 was determined using the hemagglutination test. Poly(I:C) (# tlrl-pic) was purchased from Invivogen. Anti-actin, anti-TBK-1, anti-Myc, anti-TAPE, and anti-TRIF antibodies were described previously [38]. Anti-FLAG (number F1804 and F7425) antibodies were purchased from Sigma. Anti-TLR3 (IMG-135A, IMGENEX), Anti-MAVS (A300-782A, BETHYL) and anti-V5 (GTX117997, GeneTex) antibodies were used for Western blotting. TheAnti-Enterovirus 71 Antibody (MAB979) was purchased from Merck Millipore. MG132 was purchased from Sigma. Z-Val-Ala-DL-Asp (OMe)-fluoromethylketone (Z-VAD-FMK) (N-1560) was purchased from Bachem.

### 2.3. Plasmids

Human MDA5 (hMDA5, accession number BC111750), which was obtained from Open Biosystems, was cloned into a pcDNA4/HisMax-TOPO vector (Invitrogen) [40]. FLAG-EV-A71 2A and FLAG-EV-A71 3C were kindly provided by S. H. Kung (National Yang-Ming University, Taiwan) [41,42]. pcDNA3.1-V5-His A-IRES-EV-A71 2A and pcDNA3.1-V5-His A-IRES-EV-A71 2A C110A were provided by S. Y. Lo (Tzu Chi University, Taiwan) [43]. Other plasmids were kindly provided as follows: ISRE-Luc by R. Lin (McGill University), IFN-β-Luc by K. Fitzgerald (University of Massachusetts Medical School, USA), FLAG-RIG-I by T. Fujita (Kyoto University, Japan), and FLAG-hTLR3 (Addgene plasmid 13084) by R. Medzhitov (Yale University, USA). EV-A71 2A was amplified by PCR and cloned into a pcDNA6/Myc-His vector.

### 2.4. RNA Interference

Control small interfering RNA (siRNA designed to target GFP) and siRNAs targeting TAPE, TRIF, and MAVS/IPS-1 were described previously [38,40]. Mouse TLR3-1 siRNA pool (sc-36685) was purchased from Santa Cruz Biotechnology. Mouse TLR3-2 siRNA pool (GS142980) and Human TLR3 siRNA pool were purchased from QIAGEN. HEK293 cells seeded onto 24-well plates (5 × 10^4^ cells per well) were transfected with indicated siRNAs (20 nM) by RNAiMAX (Invitrogen) for 48 h. The cells were used for reporter assays or ELISA. BMMs cells (4 × 10^5^) were transfected respectively with indicated siRNA (25 pmol) for the control, TLR3, or MAVS by electroporation using a microporator (1300 V/30 ms/1 pulse, Neon Invitrogen). The transfected cells were incubated for 72 h. Human MoDCs were transfected with 25 nM siRNA with transfection reagent DF4 (Dharmacon) according to the manufacturer’s protocol. The transfected cells were incubated for 72 h. Subsequently, they were infected with EV-A71/MP4 or EV-A71/4643 for 6 h.

### 2.5. Luciferase Reporter Assay and ELISA

HEK293 cells seeded on 24-well plates (7 × 10^4^ cells per well) were transfected with a reporter plasmid (e.g., IFN-β-Luc or ISRE-Luc) with an internal control plasmid (pRL-TK) and the indicated plasmids by lipofectamine 2000. An empty vector (pcDNA6.0/Myc-His) was used to equalize the total amount of plasmids. After 24 h, the cells were infected with EV-A71 or treated as indicated stimulants. Heat-inactivated EV-A71 was derived from the incubation of live EV-A71 at 65 °C for 30 min. For an influenza virus infection, the cells were washed with PBS, and then infected with IAV (WSN) at the indicated titers in DMEM containing 2% bovine albumin. After indicated treatments, the activities of firefly and Renilla luciferases in lysates were measured using the Dual-Luciferase assay kit (Promega) according to the manufacturer’s instructions. The firefly luciferase activity was normalized by the Renilla luciferase activity, and the fold induction of each sample was calculated relatively to a control sample. The supernatants from treated cells were collected and analyzed for IFN-β and IFN-β inducible protein 10 (IP-10), and regulated, on activation, for normal T-cell expressed and secreted (RANTES) production by ELISA. The ELISA kits were used in the study, including human IFN-β (PBL), human IP-10 (R&D Systems), and human RANTES (R&D Systems). Values represent the mean ± standard error of the mean (SE) of the duplicated samples. Data are representative of two or three experiments.

### 2.6. RT-PCR and Quantitative RT-PCR

Total RNA was extracted using RNAzol (Molecular research center), and cDNA was synthesized using a capacity cDNA Reverse Transcription Kit (Applied Biosystems). Target genes were amplified using PCR with gene-specific primers as follows: Human TLR3, sense 5’-AAATTGGGC AAGAACTCACAGG-3’ and antisense 5’-GTGTTTCCAGAGCCGTGCTAA-3’; Human β-Actin, sense 5’-AAGGAGAAGCTGTGCTACGTCGC-3’ and antisense 5’-AGACAGCACTGTGTTGGC GTA CA-3’; EV-A71 sense: 5’-GTGGCAGATGTGATTGAGAG-3’ and antisense 5’-GTTATGTCTATGTCCCAGTT-3’. A quantitative RT-PCR analysis was performed with the Applied Biosystems StepOne and SYBR Green system (Applied Biosystems). The presented data were normalized to β-actin or mL32. The mouse IFN-β primers were described previously [40]. Mouse IL-6 sense: 5’-GTAGCTATGGTACTCCAGAAGAC-3’ and antisense 5’-ACGATGATGCACTTGCAGAA-3’, human TLR3 primer sense: 5’-GATCAGATCTCAGCTTTGCCATGTTTGG-3’ and antisense 5’-ACGTGAATT CTGTTGGATGACTGCTAGCCTTTCC-3’, mouse TLR3 sense: 5’-TGGTTGGGCCACCTAGAAGTA-3’ and antisense 5’- TCTCCATTCCTGGCCTGTG-3’, mouse mL32 sense: 5’-AAGCGAAACTGGCGGAAAC-3’ and antisense 5’-TAACCGATGTTGGGCATCAG-3’ and human actin sense: 5’- CATGTACGTTGCTATCCAGGC-3’and antisense 5’- CTCCTTAATGTCACGCACGAT-3’. The following primers were used for cloning the indicated genes: EV-A71 2A using forward 5’-CTGGCTAGTTAAGCTATGGGGAAATTTGGACAGC-3’ and 5’- CCCTCTAGACTCGAGCTGCTCCATGGCTTCATC-3’.

### 2.7. Preparation and Enzyme Treatment of EV-A71-Infected RNA

HEK293 cells seeded on 6 cm plates (8 × 10^5^ cells per plate) were left either uninfected or infected by EV-A71 with 1 moi for 20 h. Total RNA was extracted from these cells using TRIzol (Invitrogen) according to the manufacturer’s instructions, and then treated with DNase (Promega). Total RNA derived from uninfected HEK293 cells through this process was termed “control RNA”. Likewise, total RNA derived from EV-A71-infected HEK293 cells was termed “EV-A71-infected RNA”. To digest double-stranded RNA, 4 µg of total RNA was treated with RNaseIII (Epicentre) at the indicated concentration at 37 °C for 30 min. To remove the 5’-triphosphate moiety, 4 µg of total RNA was digested with shrimp alkaline phosphatase (SAP) (Promega) at the indicated concentration at 37 °C for 15 min, and then at 65 °C for 15 min for heat inactivation. In the RNA experiment, the indicated RNA was transfected with DOTAP liposome (Roche) according to the manufacturer’s instructions. 

### 2.8. Plaque Assay

HEK293T cells seeded on 24-well plates were transfected with a control vector (pcDNA6.0/Myc-His) or FLAG-hTLR3 for 48 h. These cells were infected with EV-A71 for 20 h. Virus yield in cultured supernatants were collected for plaque assays using Vero cells. Briefly, Vero cells seeded on 12-well plates (1 × 10^5^ per well) were infected with serial dilutions of the cultured supernatants for 1 h, and then were overlaid with DMEM containing 2% FBS and 1% methylcellulose (Sigma). Four days after infection, the cells were stained with crystal violet solution, and plaques were counted. 

### 2.9. In Vitro Cleavage Assay

HEK293T cells were transfected with a hTLR3-FLAG for 48 h. Cells were collected into the RIPA lysis buffer without protease inhibitors. Lysates were incubated with the Anti-FLAG antibody for immunoprecipitation. In vitro transcription/translation of the pcDNA6.0/Myc-His plasmid bearing EV-A71 2A was carried out using the TnT Quick Coupled Transcription/Translation System (Promega) system. EV-A71 2A protease was purified by Ni-Agarose. Purified EV-A71 2A protease was incubated with IP lysates in the cleavage reaction buffer (20 mM Hepes, pH 7.4, 150 mM KOAc, 1 mM DTT) at 37 °C for 4 h.

### 2.10. Statistical Analysis

Statistical analyses were performed using unpaired, two-tailed, Student’s *t*-tests. The P value below 0.05 was considered to be statistically significant.

## 3. Results

### 3.1. TLR3 is Involved in Detecting EV-A71 Infection to Induce Type I IFN Antiviral Responses

**Figure 1:**
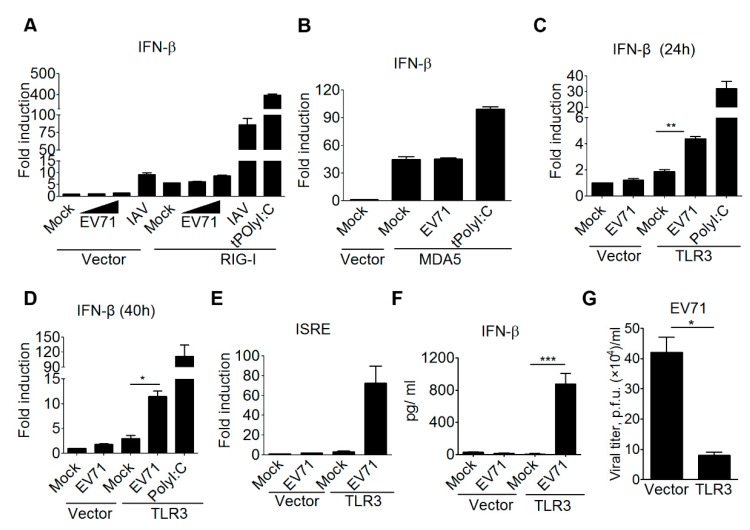
TLR3 detects EV-A71 infection to trigger type I IFN-mediated antiviral immunity. (**A**) HEK293 cells were transfected with an IFN-β luciferase reporter plasmid, plus either a control vector (pcDNA6.0) or a Flag-tagged RIG-I construct. After 24 h, the transfected cells were either left untreated, treated with poly(I:C) (0.2 µg/mL) transfection, or infected with an influenza A virus (IAV/WSN/33, 50 HA/well) or EV-A71 (4643 strain, MOI, 0.1 and 1). After another 40 h, the treated cells were harvested to analyze the IFN-β promoter activity. The EV-A71 used for most experiments was the 4643 strain, unless otherwise indicated. (**B**) Similar to Panel A, His-tagged MDA-5 was used to assess the IFN-β promoter activity in transfected HEK293 cells upon EV-A71 (MOI, 0.1) infection. (**C**–**E**) HEK293 cells were transfected with either a control vector or a Flag-tagged TLR3 plasmid with an IFN-β (**C**,**D**) or an ISRE (**E**) luciferase reporter plasmid. After 24 h, the transfected cells were either left untreated, treated with poly(I:C) (50 µg/mL), or infected with EV-A71 (MOI, 0.1). After another 24 h (**C**) or 40 h (**D**,**E**), the treated cells were harvested for luciferase assays. (**F**) HEK293 cells were transfected with either a control vector or Flag-tagged TLR3. After 24 h, the transfected cells were either left untreated or infected with EV-A71 (MOI, 0.1). After another 40 h, the supernatants were collected for ELISA. (**G**) HEK293T cells were transfected with a control vector or Flag-tagged TLR3 for 48 h, and then were infected with EV-A71 (MOI, 1) for 20 h. Viral titers in the supernatants were measured using plaque assays. * *p* < 0.05, ** *p* < 0.005, and *** *p* < 0.0005, Student’s *t*-test. Data are representative of at least two or three independent experiments.

Previous research revealed that MDA5 is able to detect EV-A71 RNA in HeLa cells and mouse embryonic fibroblasts, while Trif signaling is critical to restrain EV71 replication in a colon cancer cell line HT-29 [23,24,44]. These findings suggest that the interactions between EV71 and the host innate immune system is complicated, and the detection of EV71 infection by RNA sensing pathways could be cell context-dependent. Further, we should be cautious that some cancer and EV-A71-permissive cell lines often carry genetic mutations in innate immune pathways and type I IFN-mediated antiviral responses. Thus, to gain further insights into how mammalian RNA sensing pathways detect EV71 infection, we examined several viral RNA sensors, including TLR3, RIG-I, and MDA5, to induce type I IFN production upon EV-A71 infection in the same cellular context. For this purpose, the reconstitution experiment using a HEK293 cell line was conducted for the gain-of-function analysis for the following advantages. The HEK293 cell line expresses the low to undetectable level of RIG-I and other viral nucleic acid sensors, and its innate immune pathways and antiviral responses are functional once the specific RNA or DNA sensor is ectopically expressed in it [45,46]. Also, HEK293 cells are susceptible for various viral infections, including EV-A71. By this approach, our results first showed that the EV-A71 infection failed to induce IFN-β promoter activation in HEK293 cells, while an influenza A virus (IAV) infection slightly induced IFN-β promoter activation in HEK293 cells (Figure 1A). This IAV-induced, low IFN-β promoter activation is likely due to the low endogenous RIG-I expression in HEK293 cells [46]. Ectopic expression of RIG-I in HEK293 cells led to substantial IFN-β promoter activation in response to IAV infection or a double-stranded RNA (dsRNA) analog poly(I:C) transfection (Figure 1A). However, these RIG-I-transfected HEK293 cells failed to induce IFN-β promoter activation in response to EV-A71 infection (Figure 1A). Likewise, ectopic expression of MDA5 slightly increased the activity of the IFN-β promoter in HEK293 cells. Poly(I:C) transfection further enhanced MDA5-mediated IFN-β promoter activation, whereas EV-A71 infection failed to do so (Figure 1B). Of note, ectopic expression of TLR3 in HEK293 cells resulted in the activation of the IFN-β promoter in response to poly(I:C) stimulation or EV-A71 infection for different times (Figure 1C,D). EV-A71 infection was also shown to induce the activation of the interferon-stimulated response element (ISRE) promoter, which is a downstream event of type I IFN induction, in a TLR3-dependent manner (Figure 1E). In addition to the promoter-driven reporter assay, ELISA was also used to confirm this regulation in terms of cytokine production. The ELISA experiments also showed that EV-A71 infection induced IFN-β cytokine production from HEK293 cells in the presence of TLR3 (Figure 1F). Next, we assessed whether expression of TLR3 in HEK293T cells could confer antiviral immunity against EV-A71 infection. Plaque assays were conducted to measure the viral titer from EV-A71-infected HEK293T cells expressing either TLR3 or a control vector. Our results showed that ectopic expression of TLR3 significantly lowered the EV-A71 viral titer in HEK293T cells (Figure 1G). Together, these data support a critical role for TLR3 in detecting EV-A71 infection to trigger type I IFN-mediated antiviral immunity. 

### 3.2. Double-Stranded Viral RNA Derived from EV-A71 Replication is Essential for TLR3 Detection

**Figure 2:**
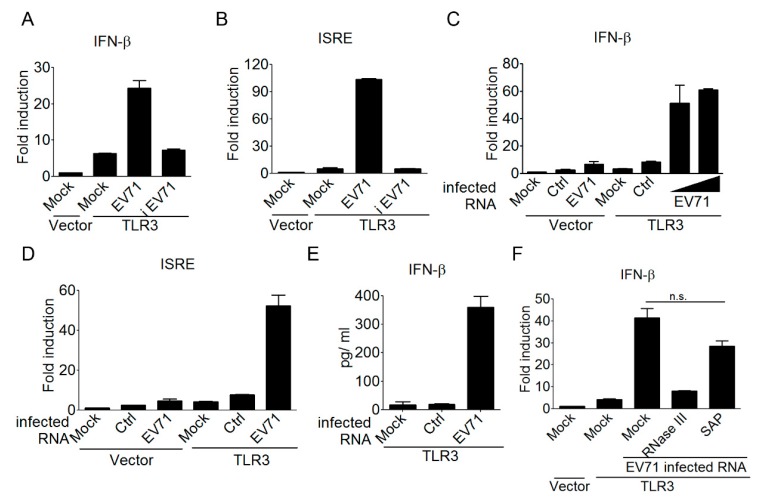
EV-A71-derived, double-stranded RNA is a ligand for TLR3 detection. (**A**–**D**) HEK293 cells were transfected with either a control vector or Flag-tagged TLR3, plus an IFN-β reporter plasmid (**A**,**C**) or an ISRE reporter plasmid (**B**,**D**). After 24 h, the transfected cells were either left untreated or infected with EV-A71 (MOI, 0.1) or heat-inactivated EV-A71 (iEV-A71) (MOI, 0.1). After another 40 h, the treated cells were harvested to analyze the IFN-β or ISRE promoter activity (**A**,**B**). Control RNA (ctrl, 2 µg/mL) and EV-A71-infected RNA (two doses, 1 and 2 µg/mL) harvested from HEK293 cells and EV-A71-infected HEK293 cells, respectively, were used to stimulate the transfected cells by DOTAP lipofection. After another 20 h, the treated cells were harvested to analyze the IFN-β or ISRE promoter activity (**C**,**D**). (**E**) HEK293 cells were transfected with Flag-tagged TLR3. Control RNA (1 µg/mL) and EV-A71-infected RNA (1µg/mL) were used to stimulate the transfected cells by DOTAP lipofection. After another 20 h, IFN-β in the supernatants was measured using ELISA. (**F**) Like Panel A, HEK293 cells were transfected as indicated. EV-A71-infected RNA left untreated or treated with RNase III or Shrimp Alkanine Phosphatase (SAP) was used to stimulate the transfected cells by DOTAP lipofection. Subsequently, these cells were subjected to luciferase assays. NS: not significant (Student’s *t*-test). Data are representative of at least two or three independent experiments.

Having shown the involvement of TLR3 in detecting EV-A71 infection, we next aimed to understand the underlying mechanism of this detection. TLR3 is known for detecting viral dsRNA during an RNA virus or some DNA virus (like HSV) infection [47]. Since EV-A71 is a positive ssRNA virus, we speculated that EV-A71 viral replication was required to generate viral dsRNA for TLR3 detection. Yet, TLR3 has also been shown to detect self-noncoding RNA from ultraviolet radiation-damaged keratinocytes [48]. Therefore, there is a possibility that TLR3 might be activated by self-RNA released from EV-A71-infected cells after cell death. Another possibility is that EV-A71 supernatants from preparation may have contained viral dsRNA, or other host components like danger molecules, to induce type I IFN activation. In order to clarify these issues on EV-A71, both live and heat-inactivated EV-A71 supernatants were used to infect HEK293 cells expressing TLR3, and then the activation of the IFN-β promoter was measured using reporter assays. Our results showed that only the live, but not the heat-inactivated EV-A71 supernatant was able to induce TLR3-mediated IFN-β activation (Figure 2A). Likewise, only the live EV-A71 supernatant was shown to activate the ISRE promoter in HEK293 cells in the presence of TLR3 (Figure 2B). These data suggest that a successful EV-A71 infection and replication within infected cells is a prerequisite for triggering the TLR3 signal to type I IFNs. We further determined whether viral RNA derived from EV-A71-infected cells served directly as a ligand for TLR3 detection. Total RNA species derived from the uninfected and EV-A71-infected HEK293 cells were designated control RNA and EV-A71-infected RNA, respectively, and were used to stimulate the TLR3 pathway. A cationic liposome DOTAP (N-[1-(2,3-Dioleoyloxy)propyl]-N,N,N-trimethylammonium methyl-sulfate), which delivers nucleic acids to the endolysosomal compartments [49], was used to transfect these two types of RNA samples for the purpose of TLR3 detection. Our results demonstrated that transfection of the EV-A71-infected RNA, but not the control RNA, was able to activate the IFN-β promoter and the downstream ISRE promoter in TLR3-transfected HEK293 cells (Figure 2C,D). ELISA analyses further confirmed that EV-A71-infected RNA triggered TLR3 signaling to the IFN-β promoter, leading to cytokine production (Figure 2E). It is established that viral RNA sensors might recognize different molecular signatures of viral RNA. For instance, RIG-I prefers to detect viral RNA containing the 5’ triphosphate moiety, while MDA5 detects long dsRNA lacking ribose 2’-O-methylation in the viral mRNA cap, and TLR3 prefers to detect long dsRNA [46,50,51,52,53]. Therefore, we were interested in analyzing the biochemical properties of EV-A71-infected RNA responsible for TLR3 detection. Endonuclease RNase III and alkaline phosphatase were used to digest dsRNA species and the 5’ triphosphate moiety of EV-A71-infected RNA, respectively. Subsequently, these untreated and treated EV-A71-infected RNA samples were used to test their effects on TLR3-mediated IFN-β activation. Our results showed that EV-A71-infected RNA after RNase III treatment significantly abolished the ability to trigger TLR3-mediated IFN-β activation, whereas EV-A71-infected RNA after alkaline phosphatase treatment still retained a considerable ability to induce this activation (Figure 2F). These results together suggest that viral dsRNA, generated from EV-A71 infection, is a major component for TLR3 sensing and signaling to type I IFNs. 

### 3.3. The TLR3 Signaling Pathway is Required for Triggering Type I IFN Response to EV-A71 Infection 

**Figure 3:**
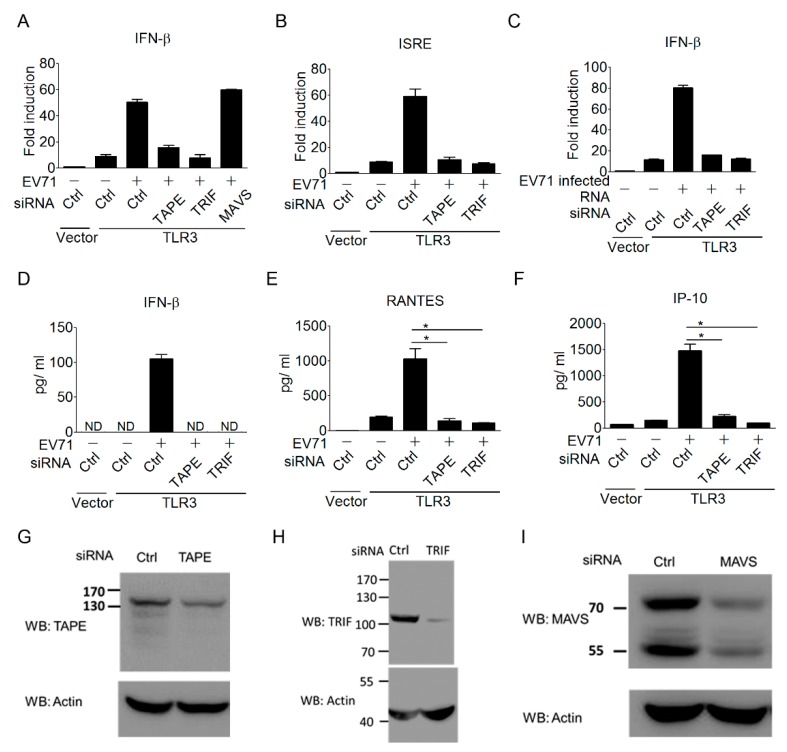
TLR3 downstream signaling is essential for triggering a type I IFN response to EV-A71 infection. (**A**,**B**) HEK293 cells were treated with the indicated siRNAs targeting Trif, TAPE, and MAVS for 48 h, and the cells were subsequently transfected with Flag-tagged TLR3 and an IFN-β (**A**) or ISRE (**B**) reporter plasmid. These cells were infected with EV-A71 (MOI, 0.1) for 40 h, and were then harvested for luciferase assays. (C) Like panel A, EV-A71-infected RNA was used to stimulate cells by DOTAP lipofection, and then the IFN-β promoter activity was measured using luciferase assays. (**E**,**F**) HEK293 cells were treated as in Panel B, with no transfection of the ISRE reporter plasmid. Supernatants from the treated cells were measured using ELISA for the production of IFN-β (**D**), RANTES (**E**), and IP-10 (**F**). (**G**–**I**) Western blot analyses of endogenous TAPE (**G**), TRIF (**H**), and MAVS (**I**) in HEK293 cells treated with indicated siRNAs or control siRNA. ND means “not detected.” * *p* < 0.05, Student’s *t*-test. Data are representative of at least two or three independent experiments.

To further confirm the importance of the TLR3 signaling pathway in triggering type I IFN antiviral responses to EV-A71 infection, we next investigated the contribution of the TLR3 downstream regulators in linking TLR3 to type I IFN induction during EV-A71 infection. Trif, also known as Ticam-1, is a key adaptor linking TLR3 and TLR4 to type I IFN induction [54,55]. TBK1-Associated Protein in Endolysosomes (TAPE), also known as CC2D1A/Freud-1/Aki-1, is a recently identified innate immune regulator involved in the TLR3, TLR4, and RLR pathways [38,40]. Thus, we assessed the involvement of Trif and TAPE in coupling TLR3 signaling to type I IFN induction during EV-A71 infection. Results from the siRNA approach showed that TLR3-mediated IFN-β activation upon EV-A71 infection was obviously impaired by the silencing of Trif or TAPE (Figure 3A). In contrast, the silencing of MAVS, a key RLR signaling downstream regulator, did not impair the activation of the IFN-β promoter during EV-A71 infection (Figure 3A). Of note, the knockdown effect of Trif or TAPE was further extended to block TLR3-mediated ISRE promoter activation during EV-A71 infection (Figure 3B). In addition to EV-A71 infection, EV-A71-infected, RNA-induced TLR3 signaling to IFN-β activation was also abolished by the silencing of Trif or TAPE (Figure 3F). Again, ELISA was used to confirm this regulation in terms of cytokine production. The ELISA results showed that silencing of Trif or TAPE impaired TLR3-mediated cytokine production of IFN-β, RANTES, and IP-10 during EV-A71 infection (Figure 3D–F). The knockdown effects of siRNAs on TAPE, Trif, and MAVS have been demonstrated in previous studies [38,55]. The knockdown effects of these siRNAs in the current work were confirmed again by immunoblotting (Figure 3G–I). These results reinforce the importance of the TLR3 signaling pathway in linking the detection of EV-A71 to trigger antiviral immunity. 

### 3.4. TLR3 Detects EV-A71 Infection To Induce Type I IFN Response in Primary Myeloid Cells

**Figure 4:**
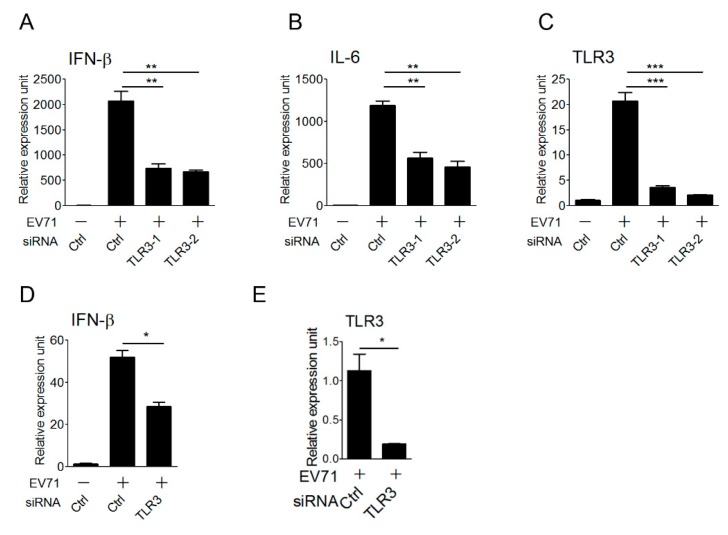
TLR3 senses EV-A71 infection in primary macrophages and dendritic cells. (**A**–**C**) Mouse bone marrow-derived macrophages (BMMs) transfected with control siRNA or two different TLR3 siRNAs were infected with EV-A71 (MP4 strain, MOI, 3) for 6 h, and then mRNA expression levels of *Ifnb* (**A**), *Il6* (**B**), and *Tlr3* (**C**) genes were measured using qRT–PCR. ** *p* < 0.005 and *** *p* < 0.0005 (Student’s *t*-test). Data are representative of at least three independent experiments. Expression is normalized to *ml32* and set at 1 in unstimulated cells with control siRNA. (**D**,**E**) Human monocyte-derived dendritic cells (MoDCs) transfected with control siRNA or TLR3 siRNA were infected with EV-A71 (MOI, 3) for 6 h, and then mRNA expression levels of *Ifnb* (**D**) and *Tlr3* (**E**) genes were measured using qRT-PCR. * *p* < 0.05 (Student’s *t*-test). NS stands for “not significant.” Data are representative of at least three independent experiments. Expression is normalized to *actin* and set at 1 in unstimulated cells with control siRNA.

Antigen-presenting cells, like macrophages and dendritic cells, play an important role in the innate immune defense against viral infection, and are also linked to the activation of adaptive immunity. These cells express endogenous TLR3 and other viral nucleic acid sensors to sense viral nucleic acids during viral infection, thereby triggering type I IFN antiviral responses. After demonstrating the involvement of TLR3 in detecting EV-A71 infection in the reconstituted HEK293 cells, we attempted to further confirm the role of endogenous TLR3 in primary antigen-presenting cells in the detection of EV-A71 infection to trigger type I IFN activation. To this purpose, the small interfering RNA approach was used for the loss-of-function analysis. Mouse bone marrow-derived macrophages (BMMs) were first transfected with the control and two sets of TLR3 siRNAs, and transfected BMMs were infected with EV-A71 for 6 hours and then subjected to quantitative real-time PCR (qPCR). Our results showed that TLR3 knockdown in BMMs significantly impaired the expression of IFN-β and IL-6 cytokine genes upon EV-A71 infection (Figure 4A,B). The silencing effect of TLR3 on mouse BMMs was confirmed by the decrease in the TLR3 mRNA level even though TLR3, as one of ISGs, was also upregulated upon EV-A71 infection (Figure 4C). Likewise, silencing of TLR3 in human monocyte-derived dendritic cells (MoDCs) led to lower IFN-β and TLR3 mRNA expression in response to EV-A71 infection (Figure 4D,E). Together, these data support a critical role for TLR3 in primary innate immune cells to induce type I IFNs upon detecting EV-A71 infection.

### 3.5. EV-A71 Infection Decreases the TLR3 Protein Level

**Figure 5:**
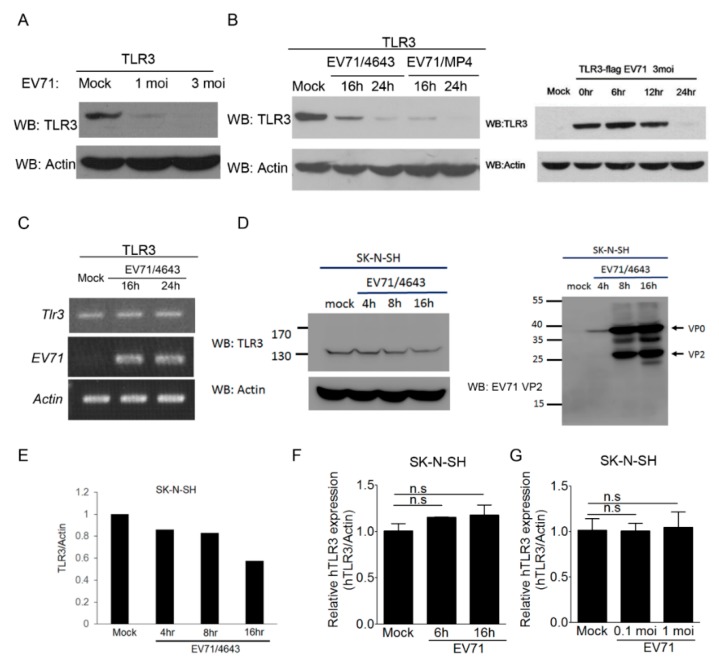
The TLR3 protein level is decreased upon EV-A71 infection. (**A**) HEK293 cells transfected with Flag-tagged TLR3 were left untreated or infected with EV-A71 (MOI, 1 and 3). After 24 h, the treated cells were harvested for the Western blot analysis of TLR3 protein levels. (**B**) Similar to panel A, EV-A71 (4643 strain, MOI, 3) and EV-A71 (MP4 strain, MOI, 1) were used to infect TLR3-transfected HEK293 cells for the indicated times. These cells were then harvested for the Western blot analysis of TLR3. (**C**) Like Panel B, the treated cells were harvested at the indicated times to analyze the mRNA levels of *Tlr3*, *EV-A71*, and β-*Actin* by RT-PCR. (**D**,**E**) SK-N-SH cells were left untreated or infected with EV-A71 (4643 strain, MOI, 0.2) for 4 h, 8h, and 16 h, and the cells were harvested for the Western blot analysis of the endogenous TLR3 protein level. (**F**) SK-N-SH cells were left untreated or infected with EV-A71 (4643 strain, MOI, 0.1), and the cells were harvested at the indicated times (6 h and 16 h) to analyze the mRNA levels of *Tlr3* and β-*Actin* by qRT-PCR. (**G**) SK-N-SH cells were left untreated or infected with EV-A71 (4643 strain, MOI, 0.1 and 1) for 6 h, and the cells were harvested to analyze the mRNA levels of *Tlr3* and β-*Actin* using qRT-PCR. NS stands for “not significant” (Student’s *t*-test). Data are representative of at least two or three independent experiments.

Viruses have constantly evolved to evade the host immune attacks for their propagation. Recent studies indicated that enteroviruses, like the poliovirus, coxsackievirus, and EV-A71, might target the viral RNA-sensing pathways to counteract host antiviral responses through viral proteases [29,33]. Notably, Trif/Ticam has been shown to be a target of EV-A71 3C protease [29,44]. Thus, we were interested in assessing whether EV-A71 might target TLR3 for innate immune evasion. TLR3-transfected HEK293 cells were used for EV-A71 infection, and the TLR3 protein level was measured using immunoblotting. As shown in Figure 5A, the TLR3 protein level was decreased after EV-A71 infection in a dosage-dependent manner. Results from kinetic analyses further demonstrated that EV-A71 infection led to the decrease of the TLR3 protein level in a time-dependent manner (Figure 5B). Similarly, a mouse-adapted strain EV-A71/MP4 decreased the TLR3 protein level in a more profound manner (Figure 5B). In light of previous studies [29,33], we speculated that the decrease of TLR3 protein might be mediated by EV-A71 proteases. Before testing this idea, we first conducted reverse transcription-PCR analyses to rule out the possibility that the mRNA level of TLR3 was reduced during EV-A71 infection. Our results showed that the TLR3 mRNA levels from transfected HEK293 cells exhibited no apparent change before and after EV-A71 infection (Figure 5C), suggesting that EV-A71-induced TLR3 downregulation was not on the transcriptional level. It is critical to point out that the TLR3 expression construct used in this study was driven by the CMV promoter instead of the endogenous ISRE promoter. Thus, it is different from the case of endogenous TLR3 mRNA upregulation shown in Figure 4C and other studies [44]. Next, we attempted to confirm whether this phenomenon also occurred at the endogenous TLR3 level in an EV-A71-permissive cell line SK-N-SH, which is a human neuroblastoma cell line susceptible to EV-A71 infection [56]. As shown in Figure 5D,E, the endogenous TLR3 protein level in SK-N-SH cells was also decreased after EV-A71 infection in a time-dependent manner. Consistent with a previous report [39], the replicative cycle of EV-A71 in SK-N-SH cells was completed within 16 hours (Figure 5D). Furthermore, results from qPCR analyses showed that the endogenous TLR3 mRNA levels in SK-N-SH cells exhibited no significant reduction at different time points or at different doses of EV-A71 infection (Figure 5F,G). It is unclear why the endogenous TLR3 mRNA level in SK-N-SH cells was not upregulated upon EV-A71 infection. A previous study indicated that type I IFN receptor-signaling in another EV-A71-permissive RD cell line was impaired during EV-A71 infection because EV-A71 2A targeted the IFNAR1 protein for degradation [35]. It is likely that a similar phenomenon could occur in SK-N-SH cells. Nevertheless, these data provide the first evidence that EV-A71 infection decreases the TLR3 protein level in infected cells. 

### 3.6. EV-A71 Protease 2A Targets TLR3 to Inhibit Type I IFN Induction 

**Figure 6:**
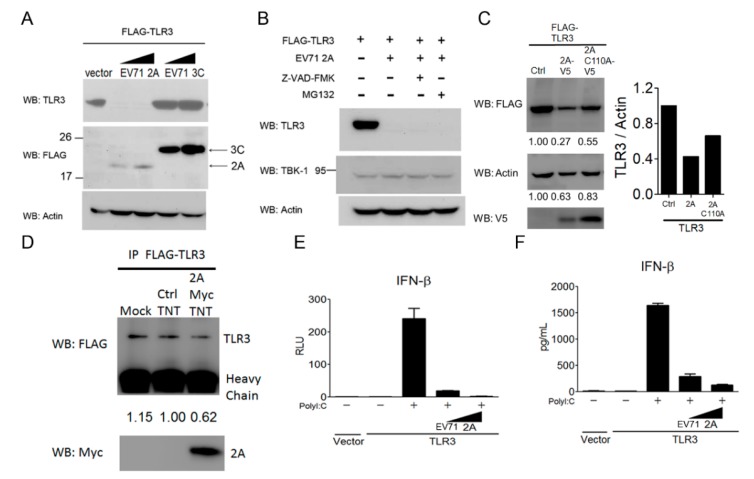
EV-A71 protease 2A targets TLR3 to inhibit type I IFN induction. (**A**) HEK293 cells were transfected with Flag-tagged TLR3 alone or together with increasing amounts of either Flag-tagged EV-A71 2A or Flag-tagged EV-A71 3C. After 20 h, the transfected cells were harvested for the Western blot analysis using the indicated antibodies. (**B**) HEK293 cells were transfected with Flag-tagged TLR3 alone or together with Flag-tagged EV-A71 2A. After 4 h, the transfected cells were treated with a pan-caspase inhibitor Z-VAD-FMK (100 μM) or a proteasome inhibitor MG132 (20 μM). After another 16 h, the treated cells were harvested for the Western blot analysis using the indicated antibodies. (**C**) HEK293 cells were transfected with Flag-tagged TLR3 alone or together with V5-tagged EV-A71 2A or V5-tagged EV-A71 2A C110A. After 48 h, the transfected cells were harvested for the Western blot analysis using the indicated antibodies. (**D**) 293T cells were transfected with FLAG-TLR3. Cell lysates were subjected to the immunoprecipitation and incubated with purified EV-A71 2A protease at 37 °C for 4 h; treated cell lysates were then subjected to Western blotting. (**E**,**F**) HEK293 cells were transfected with an IFN-β luciferase reporter together with Flag-tagged TLR3 only or with increasing amounts of Flag-tagged EV-A71 2A. After 24 h, the transfected cells were left untreated or were treated with poly(I:C) (50 µg/ml). After another 20 h, the treated cells were harvested to analyze the IFN-β promoter activity using luciferase assays (**D**) or IFN-β cytokine production using ELISA (**E**). Data are representative of at least two or three independent experiments.

Since EV-A71 2A and 3C proteins are viral proteases shown to target several innate immune regulators for degradation [28,29,30,33,34], we next explored whether these two viral proteases were involved in the downregulation of TLR3 in mammalian cells. HEK293 cells were co-transfected with TLR3 in combination with a control vector, EV-A71 2A or 3C. The protein level of TLR3 in the transfected cells was examined using immunoblotting. Our results showed that the TLR3 protein level in transfected cells significantly decreased in the presence of EV-A71 2A, but not EV-A71 3C (Figure 6A, the upper panel). The protein expression of EV-A71 2A and EV-A71 3C was confirmed by immunoblot analyses (Figure 6A, the middle panel). Since EV-A71 2A and 3C have been shown to induce EV-A71-infected cell apoptosis through the cleavage and activation of cellular caspases [57,58], we then tested whether the caspase pathway or the proteasome pathway was involved in EV-A71 2A-mediated TLR3 downregulation. To this end, a pan-caspase inhibitor Z-VAD-FMK and a proteasome inhibitor MG132 were used to block EV-A71 2A-induced TLR3 downregulation in HEK293 cells. Our results revealed that neither Z-VAD-FMK nor MG132 was able to block EV-A71 2A-induced TLR3 down-regulation in HEK293 cells (Figure 6B, the upper panel), suggesting that EV-A71 2A decreases the TLR3 protein level in a manner independent of caspase- and proteasome-mediated degradation. TBK1 is a key protein kinase linking several viral RNA- and DNA-sensing pathways to type I IFN induction [59]. Its endogenous protein level in HEK293 cells showed no apparent change before and after EV-A71 2A expression (Figure 6B, the middle panel), suggesting a selective effect of EV-A71 2A on TLR3. To further assess the effect of EV-A71 2A protease activity on the downregulation of TLR3, an EV-A71 2A-C110A mutant was used for our analysis. Our results showed that expression of the EV-A71 2A-C110A mutant did not lead to a decrease in TLR3 as significant as that of the expression of EV-A71 2A (Figure 6C), suggesting that EV-A71 2A protease activity is critical for TLR3 degradation. To explore whether EV-A71 2A protease was responsible for the direct cleavage of TLR3, we performed in vitro cleavage assays. EV-A71 2A protease with Myc-His tag was synthesized using in vitro transcription and the translation expression system, and purified by nickel beads. Purified EV-A71 2A protease was added to TLR3 immunoprecipitation lysates for in vitro cleavage. Our results showed that the protein level of TLR3 partially decreased in the presence of EV-A71 2A protease (Figure 6D). Next, we attempted to confirm the effect of EV-A71 2A on the TLR3 signaling pathway to type I IFN induction. The results from the reporter assays showed that TLR3-mediated IFN-β promoter activation by poly(I:C) was significantly diminished in the presence of EV-A71 2A (Figure 6E). Likewise, this phenomenon was extended to the cytokine production of IFN-β (Figure 6F). In combination with Figure 5, these results suggest that EV-A71 2A protease targets TLR3 during EV-A71 infection via a post-transcriptional and translational manner. 

## 4. Discussion

Since its initial discovery in the 1970s, EV-A71 has become an important infectious agent causing HFMD and neurological disorders in the Asia-Pacific region, as well as other areas. The pathogenesis of EV-A71 infection is still poorly understood. Like other infectious diseases, both viral and host factors contribute to the outcome of EV-A71-mediated diseases. Identification of SCARB2 and PSGL-1 as receptors for EV-A71 infection paves the avenue toward understanding the interaction between EV-A71 and host cells [5,6]. Furthermore, it is also important to understand how the host innate immune system detects EV-A71 infection at the first place to trigger antiviral immunity, and meanwhile how EV-A71 subverts host antiviral responses to successfully establish its infection. Although cumulative evidence reveals that EV-A71 targets several viral RNA sensing pathways, the detailed mechanisms by which viral RNA sensors detect EV-A71 infection to elicit type I IFN antiviral responses are not yet well understood. Our current work provides the first evidence to support a critical role for the TLR3 pathway in detecting and defending against EV-A71 infection. Gain-of-function analyses showed that ectopic expression of TLR3, but not RIG-I or MDA5, in HEK293 cells led to type I IFN-mediated antiviral immunity against EV-A71 infection. Loss-of-function analyses further confirmed the importance of TLR3 and its downstream regulators, Trif and TAPE, in detecting EV-A71 infection and signaling to type I IFN activation. The importance of TLR3 in detecting EV-A71 infection was also confirmed in human primary dendritic cells and mouse primary macrophages. Furthermore, our results suggest that EV-A71 protease 2A targets TLR3 for degradation to impair TLR3 sensing and signaling. Together, these data demonstrate the importance of TLR3 in mounting antiviral defenses against EV-A71 infection by induction of type I IFNs and inflammatory cytokines. 

It is known that endosomal TLRs (TLR3 and TLR7), cytosolic RLRs (RIG-I and MDA5), and other cytosolic RNA sensors play key roles in the recognition of RNA virus infection to trigger type I IFN antiviral responses [19,20]. Using reconstitution experiments, we examined the ability of TLR3, RIG-I, and MDA5 to detect EV-A71 infection and induce type I IFN antiviral immunity in HEK293 cells. Our data showed that only TLR3 detected live EV-A71 infection to trigger type I IFN activation (Figure 1 and Figure 2). In addition to the reconstitution experiments, our data from the siRNA knockdown analyses further corroborated the function of endogenous TLR3 in triggering IFN-β expression in primary macrophages and dendritic cells upon EV-A71 infection (Figure 4). Our results from real-time PCR analyses showed that TLR3 was critical for detecting EV-A71 infection to induce IFN-β and IL-6 mRNA expression at an early time-point (6 h) in primary immune cells (Figure 4), suggesting that TLR3 plays a crucial role in the early detection of EV-A71 infection. This finding extends the functional role of TLR3 in detecting EV-A71 infection from HEK293 cells to primary innate immune cells. Our biochemical analyses provided molecular insights into understanding TLR3-mediated detection and downstream signal transduction during EV-A71 infection. Consistent with the prevailing notion that TLR3 is involved in sensing viral dsRNA and poly(I:C), our data indicated that TLR3 mainly detected viral dsRNA from EV-A71 replication to trigger IFN-β activation (Figure 2F). This kind of viral dsRNA is likely to be generated in the host cells during EV-A71 infection because the EV-A71 genome only contains positive ssRNA. In this regard, a rising question is how endosomal TLR3 can detect cytosolic EV-A71-derived dsRNA. Autophagy is shown to play a critical role in delivering cytosolic viral nucleic acids to endolysosomal compartments for TLR sensing [60]. Another study indicated that autophagy was required for TLR3 signaling to type I interferon activation [61]. Consistent with these findings, we also noticed that silencing an autophagy regulator, ATG5, impaired TLR3-mediated IFN-β activation upon EV-A71 infection (data not shown). However, we do not rule out a possibility that TLR3 in bystander cells may also detect EV-A71-derived dsRNA released from infected cells via the endocytic route. 

Notably, cumulative evidence from studying patients and murine models indicates that TLR3 plays an important role in protection from neurotropic virus infections, such as herpes simplex virus 1, poliovirus, EMCV, and coxsackievirus B [25,26,62,63,64,65]. We initiated this work several years ago without the knowledge of any published TLR3-IFN-EV-A71 work at that time. Key findings of the current work were first published in the first author’s dissertation [66]. During the course of conducting the current study, a study reported that TLR3 signaling in macrophages was required for invariant natural killer T (iNKT) cell activation during EV-A71 infection, and that this iNKT population played a key role in defending against EV-A71 infection in vivo [67]. Further, their work demonstrated that TLR3-deficient mice were more vulnerable to EV-A71 infection than wild-type mice [67]. However, their work did not address the role of TLR3 in defending against EV-A71 infection through type I IFN-mediated antiviral immunity. Another recent study showed that Trif signaling was critical for sensing and defending against EV-A71 infection in a colon cancer cell line HT-29 [44]. Their data are interesting and first suggest the role of the TLR3-Trif axis in detecting EV-A71 infection. However, there was no result about TLR3 in that work. The Trif adaptor is known to function downstream of TLR3 and TLR4. The effect of Trif-signaling solely on TLR3-signaling should be cautiously accounted for, since a report also indicates that EV-A71 can activate the TLR4 pathway [68]. Complementary to those previous studies, our current work clearly demonstrates the critical role of the TLR3 pathway in sensing and defending against EV-A71 infection in HEK293 cells and primary innate immune cells. Future work using TLR3 knockout mice is warranted to further confirm the importance of the TLR3-type I IFN axis in protecting from EV-A71 infection in vivo. 

MDA5 has emerged as a viral RNA sensor for detecting several picornaviruses, such as EMCV, coxsackievirus B, and the poliovirus [20,21,22]. Recent studies have indicated that MDA5 is involved in detecting EV-A71 viral RNA or infected RNA in mammalian cells [23,24]. Thus, we also tested whether EV-A71-infected RNA could be detected by cytosolic MDA5 or RIG-I in transfected HEK293 cells. Consistent with previous reports [23,24], our results from reconstitution experiments also demonstrated that MDA5 was able to detect EV-A71-infected RNA to activate the IFN-β promoter in HEK293 cells (data not shown). Similar results were observed in the case of RIG-I (data not shown). These results suggest that EV-A71-infected RNA contains viral RNA species for RIG-I and MDA5 sensing. Of note, our data suggest that live EV-A71 infection may engage with the RNA-sensing pathways in mammalian cells in a manner distinct from EV-A71-infected RNA transfection. The underlying mechanism to doing so is still unclear. One possibility is that EV-A71 might employ diverse strategies to subvert MDA5-mediated sensing or signaling to type I IFNs in HEK293 cells. In line with this idea, MDA5, IPS-1/MAVS, and other innate immune regulators have been shown to be the targets of EV-A71-derived proteases [24,34,37]. However, there is still a possibility that MDA5 may play a role in detecting EV-A71 infection in other cell types. TLR7 is another RNA sensor for detecting viral ssRNA in plasmacytoid dendritic cells (pDCs) during RNA virus infection [69,70]. Given the fact that TLR7 is unable to activate type I IFNs in the context of HEK293 cells [71], we thus did not include TLR7 in our reconstitution experiments. It would be interesting to examine whether TLR7 is implicated in sensing EV-A71 infection in pDCs.

EV-A71-encoded 2A and 3C proteases function to process a large EV-A71 multi-protein precursor to produce mature structural and non-structural proteins during viral replication [72,73]. These EV-A71 proteases have emerging roles in interacting host cellular processes to facilitate EV-A71 propagation [72,73]. Recent evidence further indicates that several innate immune sensors and regulators appear to be the targets of these EV-A71 proteases [28,29,30,33,34,35,37]. Notably, Trif/Ticam-1, a key TLR3 downstream mediator, is shown to be cleaved by EV-A71 3C protease [29]. To further add to the complexity to this scenario, our data also suggest that TLR3 could be a target of EV-A71 2A protease (Figure 5 and Figure 6). However, it remains unclear whether EV-A71 2A mediates TLR3 downregulation through direct cleavage or in an indirect manner. Based on the known EV-A71 2A cleavage motif [74], we performed the sequence analysis and found some potential cleavage sites within TLR3 for EV-A71 2A. In vitro cleavage assays were then conducted to test whether EV-A71 2A was responsible for the direct cleavage of TLR3. Our results showed a marginal effect of EV-A71 2A on in vitro cleavage of TLR3 (Figure 6D). Our data suggest that in vitro synthesized EV-A71 2A protease seems not to function as well as the cellular one from EV-A71 2A overexpression or EV-A71 infection. We speculate that in vitro synthesized EV-A71 2A might be too unstable to fully enact protease activity, and further studies are needed to determine the effect of EV-A71 2A on the cleavage of TLR3. In addition, our results showed no detectable TLR3 cleavage products after EV-A71 infection or EV-A71 2A expression, suggesting that TLR3 cleavage products may be too unstable to be further degraded in cells. Actually, similar phenomena have also been observed in previous studies [34,35]. One study demonstrates that EV-A71 2A reduces the IFNAR1 protein level during EV-A71 infection, leading to the downregulation of IFNAR signaling to ISG induction [35]. However, no cleaved IFNAR1 product was detectable in EV-A71 2A-expressed HEK293T cells [35]. Another study indicates that EV-A71 2A directly cleaves MAVS to yield MAVS cleaved bands by an in vitro assay. Yet, no cleaved MAVS band is found in EV-A71 2A-expressed mammalian cells, while MAVS is decreased [34]. Further studies are also warranted to explore the dynamic interplay between EV-A71 2A-mediated counteract and TLR3-mediated type IFN induction, which might have a critical impact on the outcomes of EV-A71 infection. Our data suggest that this interplay could be affected by various factors, including viral strain, viral titer, and cell type (Figure 5). Our results from time course analyses indicated that TLR3 degradation caused by EV-A71 infection could occur as early as 12 h post-infection (Figure 1J), and the phenomenon became more evident after 24 h post-infection. Notably, the EV-A71/MP4 strain displayed a more potent ability to cause TLR3 degradation compared with the EV-A71/4643 strain. The underlying mechanism of this phenomenon has yet to be further explored.

**Figure 7:**
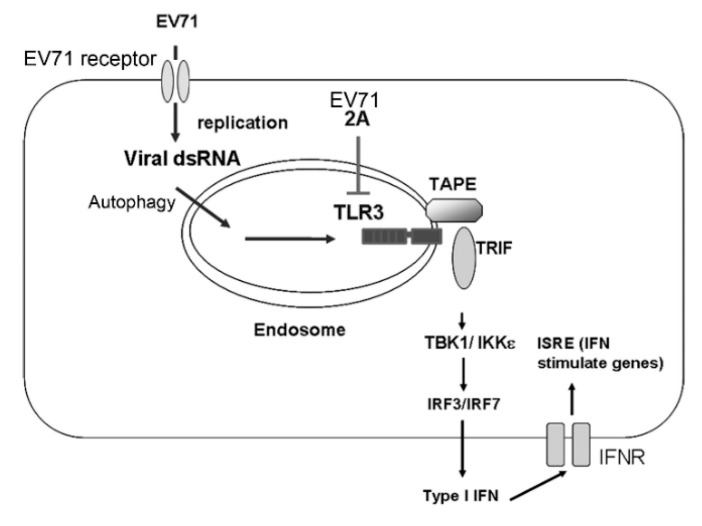
A model for the detection of EV-A71 infection by TLR3. Upon infection into host cells, EV-A71 generates dsRNA during viral replication. Endosomal TLR3 in host cells detects EV-A71 dsRNA, possibly via autophagy, to recruit the adaptor TRIF and TAPE, which further trigger signaling pathways to IFN-β activation and subsequent expression of IFN-stimulated genes (ISGs). To promote EV-A71 survival in host cells, EV-A71 uses its 2A protease to target TLR3 for degradation to counteract type I IFN-mediated antiviral responses.

Our current work has shed light on how the TLR3 pathway plays a critical role in detecting EV-A71 infection, thereby triggering type I IFN-mediated antiviral immunity (Figure 7). This could be a key step toward understanding how EV-A71 engages with the host innate immune system to initiate the first wave of innate immune responses and then affect subsequent adaptive immune responses to EV-A71 infection. In combination with previous research, our current work suggests that EV-A71 proteases may help to subvert the TLR3-Trif pathway to facilitate EV-A71 infection and replication in host cells (Figure 7). Further studies to understand this regulation may provide insights into the development of EV-A71 vaccines and the treatment of EV-A71 infection in the future.
